# National Early Warning Score for Predicting Clinical Outcome of Acute Pulmonary Embolism in Intermediate–High Risk Patients

**DOI:** 10.1055/a-2719-9061

**Published:** 2025-11-04

**Authors:** Audrey J. C. Overgaauw, Esther J. Nossent, Lilian J. Meijboom, Erik H. Serne, Yvo M. Smulders, Prabath W. B. Nanayakkara, Harm Jan Bogaard, Pieter Roel Tuinman, Frederikus A. Klok

**Affiliations:** 1 Department of Internal Medicine, Amsterdam UMC, Vrije Universiteit Amsterdam, Amsterdam, The Netherlands; 2 Department of Pulmonary Medicine, Amsterdam UMC, Vrije Universiteit Amsterdam, Amsterdam, The Netherlands; 3 Amsterdam Cardiovascular Sciences Research Institute, Amsterdam UMC, Amsterdam, The Netherlands; 4 Department of Radiology and Nuclear Medicine, Amsterdam UMC, Vrije Universiteit Amsterdam, Amsterdam, The Netherlands; 5 Department of Intensive Care Medicine, Amsterdam UMC, Vrije Universiteit Amsterdam, Amsterdam, The Netherlands; 6 Department of Medicine – Thrombosis and Hemostasis, Leiden UMC, Leiden, The Netherlands

**Keywords:** pulmonary thromboembolism, risk assessment, critical care, early warning score

## Abstract

**Background:**

Although the European Society of Cardiology (ESC) predicts mortality in acute pulmonary embolism (PE), it may overtriage the level of clinical monitoring needed. The National Early Warning Score (NEWS) is used to triage level of care in many diseases, but it is rarely reported in PE literature.

**Methods:**

In this retrospective, single-center, observational cohort study of consecutive adults with acute PE, between 2017 and 2020, we aim to assess the association between NEWS and the risk of hemodynamic (HD) deterioration or PE-related death in intermediate–high risk PE patients. The NEWS at admission and after 24 hours were determined. A baseline NEWS of ≥5 or the maximum score of a single parameter was considered an indication of high risk of our primary outcome (hemodynamic deterioration and/or PE-related mortality).

**Results:**

ESC classified 99 of 318 patients with PE as intermediate–high risk; 8 patients (8%) met the primary outcome. A total of 52 (52%) patients had an elevated NEWS and 7 of these met the primary outcome (13%), while only 1 patient with a non-elevated NEWS (2.0%) met the primary outcome (negative predictive value of 98%; 95% CI 90–98%). Sensitivity of elevated NEWS in patients with intermediate–high risk was 88% (95% CI 74–90%) and the specificity was 51% (95% CI 41–61%).

**Conclusion:**

Using NEWS in intermediate–high risk, acute PE patients may improve accuracy in identifying patients with a higher risk of adverse outcomes and may guide the decision to monitor a patient in a high-care department, especially in patients with intermediate–high risk PE.

## Introduction

A decreasing trend in pulmonary embolism (PE)-related mortality has been observed over the past two decades. 1 One of the drivers of the lower number of PE-related deaths across the European Union is advanced risk stratification that has been gradually introduced: right ventricle (RV) function and cardiac biomarkers, shown to be prognostic markers in acute PE, were incorporated into the European Society of Cardiology (ESC) and European Respiratory Society (ERS) risk stratification algorithm. 2 3 4 5 6 7 8 In short, patients are classified into a low, intermediate, or high-risk category. Those with high-risk PE require immediate reperfusion treatment while those at low risk may be treated at home. For patients with intermediate risk PE, the guideline suggests hospital admission, with close monitoring in case of a combination of signs of RV overload or dysfunction with an abnormal troponin test result indicative of ischemia, the so-called intermediate–high risk PE category. 2 3 9 However, there is a wide variation in clinical presentations within this intermediate–high risk category, with a 7-day incidence of hemodynamic deterioration or PE-related death ranging from 1 to 6% in the two largest dedicated prospective outcome trials currently available. 10 11 To admit all intermediate–high risk patients for close monitoring seem to be in contrast with modern healthcare's focus on appropriate care, defined by the World Health Organization as care that is effective, efficient, and in line with ethical principles of “fair allocation of critical care resources.” 12 Appropriate care becomes more and more important due to rising health care costs and strained resources. 13 Additional tools are necessary to improve risk stratification to better identify patients who would benefit from admission to high-care departments and possibly reduce high-care admittance of patients with a low risk of deterioration.

The National Early Warning Score (NEWS) is a general and well-validated risk score for clinical deterioration in multiple clinical settings. 14 15 16 NEWS is a widely used prognostic score in emergency, surgical, and medical departments, aimed at early identification of patients who require advanced care. We hypothesized that adding the NEWS to the current prognostic assessment of intermediate–high risk patients would improve risk stratification. This study aims to assess the association between NEWS at baseline and its course during the first 24 hours, and the risk of hemodynamic (HD) deterioration or PE-related death in intermediate–high risk PE patients.

## Methods

### Design and Setting

This is a retrospective observational cohort study performed on data from the previously published study by Overgaauw et al 17 on acute PE patients in the Amsterdam University Medical Centers, location VUmc, a tertiary university hospital in The Netherlands. Based on the ESC guideline of 2014, 2 we implemented a local protocol suggesting to consider admission to a high-care department (intensive care unit [ICU], medium care unit [MCU], or coronary care unit [CCU]) for patients with high-risk and intermediate–high risk acute PE per August 01, 2017. Clinicians could ultimately refrain from high-care admission based on individual considerations. We collected all relevant patient and outcome data from the electronic patient charts.

### Patient Selection

All consecutive adult patients with computed tomography pulmonary angiography (CTPA)-confirmed first or recurrent acute PE between August 1, 2017 and March 1, 2020 were eligible for study inclusion. Acute PE was defined as a contrast filling defect on CTPA at the subsegmental or more proximal level, or, when CTPA was not available, when there was echocardiographic evidence of RV overload/pulmonary hypertension in the absence of alternate causes for shock, where reperfusion therapy was initiated based on clinical suspicion. 18 Patients with missing data to perform an adequate risk stratification of PE severity and patients who were living abroad and consequently had no routine follow-up were excluded. The study protocol was approved by the institutional review board (registration number 2020.498) with a waiver for informed consent due to the retrospective design of the study.

### Outcomes

Primary outcome was the composite outcome of HD deterioration and/or 30-day PE-associated mortality. PE-associated mortality was defined as death by PE when clinically suspected by the treating physician or confirmed by autopsy, and HD deterioration was defined as a declining blood pressure with signs of reduced tissue perfusion, such as an altered mental state or raised serum lactate requiring rescue reperfusion therapy. 3

### Data Source

Data were collected by the primary researcher, who has 8 years of training and experience in the field of acute internal medicine. The same researcher calculated NEWS and performed risk stratification. The following data were retrieved from the electronic patient files: age, gender, vital signs, indicators of shock (e.g., altered mental state and lactate), history of malignancy, and chronic heart failure or chronic lung disease for baseline characteristics. Parameters to calculate NEWS according to Table 1 were derived from nursing notes in electronic patient files. NEWS at admission and 24 hours were calculated according to the original definition of the Royal College of Physicians (RCPL) National Early Warning Score Design and Implementation Group. 14 The NEWS were not calculated during the care for the patients nor used for clinical decision-making. We used the first documented vital signs within 12 hours after first presentation to determine the first NEWS at admission. The second NEWS was calculated at 24 hours with a maximum deviation of 4 hours. In patients with one or more missing parameters to calculate NEWS, we calculated the minimum NEWS with the available parameters. We defined NEWS as high risk when total NEWS was 5 or above, or when a single parameter scored 3 points, based on recommendations of the NHS RCPL report on NEWS. 19 NEWS was deemed stable when it was the same at 24 hours, improved when it was lower at 24 hours compared with baseline, and worsened when higher at 24 hours compared with baseline.

**Table 1:** National Early Warning Score

Physiological parameters	3	2	1	0	1	2	3
Respiration rate (br/min)	≤8		9–11	12–20		21–24	≥25
Oxygen saturations (%)	≤91	92–93	94–95	≥96			
Supplemental oxygen		Yes		No			
Temperature (°C)	≤35.0		35.1–36.0	36.1–38.0	38.1–39.0	≥39.1	
Systolic BP (mmHg)	≤90	91–100	101–110	111–219			≥220
Heart rate (b/min)	≤40		41–50	51–90	91–110	111–130	>131
Level of consciousness				A			V, P, or U

Abbreviations: A, alert; b/min, beats per minute; br/min, breaths per minute; °C, degrees Celsius; mmHg, millimeters of mercury; P, response to pain; U, unresponsive; V, response to speaking.

Risk classification was performed by the primary study investigator based on the 2014 ESC/ERS guidelines, 2 using RV dysfunction on CTPA, defined as an RV/left ventricle (LV) diameter ratio >1.0, and presence of an elevated troponin T concentration. Troponin T >0.014 μg/L was considered elevated. If patients were transferred to another hospital for treatment, all relevant clinical information was retrieved.

### Statistical Analysis

SPSS version 24 (IBM Corp., Armonk, USA) was used for statistical analysis. To evaluate patient characteristics and outcomes, descriptive statistics were performed. Normally distributed variables are presented as mean (± standard deviation [SD]), and non-normally distributed variables are expressed as medians with interquartile ranges (IQR). Univariate logistic regression was used to evaluate odds ratio (OR) of the primary outcome between NEWS. Two by two contingency tables were used to evaluate the OR of the composite outcome of HD deterioration and/or 30-day PE-associated mortality and the sensitivity, specificity, negative predictive value, and positive predictive values for elevated NEWS in predicting this primary composite outcome. The Wilson-score method was used to calculate 95% confidence intervals.

## Results

### Patient Characteristics

**Fig. 1:**
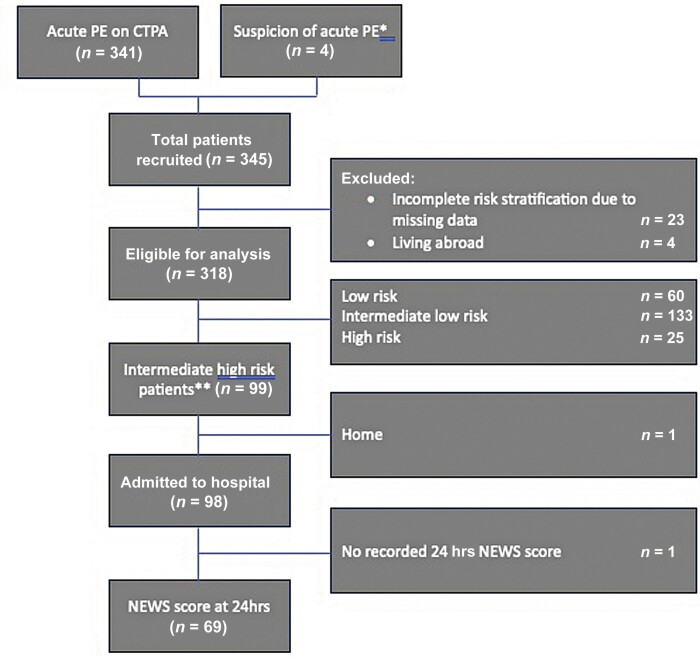
STROBE flowchart (Database of Overgaauw et al 17 ). CTPA, computerized tomography with pulmonary angiogram; *n* , number of patients; PE, pulmonary embolism; RvF, right ventricle failure; STROBE, an international, collaborative initiative of epidemiologists, methodologists, statisticians, researchers, and journal editors involved in the conduct and dissemination of observational studies with the common aim of strengthening the reporting of observational studies in epidemiology; TTE, transthoracic echocardiography. *Clinical suspicion when CTPA was not available, when there was echocardiographic evidence of RV overload/pulmonary hypertension in the absence of alternate causes for shock, where reperfusion therapy was initiated. **In all 99 intermediate–high risk patients a baseline NEWS could be calculated.

The original database consisted of 345 acute PE patients; 23 patients were excluded due to missing data to complete accurate risk classification by ESC/ERS guidelines and 4 patients were lost to follow-up ( Fig. 1 ). This resulted in a total of 318 acute PE patients in the final study cohort. Clinical characteristics of these patients are shown in Table 2 . Of 318 patients 27 (9%) were classified as high risk and 99 (31%) as intermediate–high risk patients. Of the 98 intermediate–high risk patients admitted to the hospital, 82 (84%) remained hospitalized at 24 hours. Complete NEWS at baseline could be retrieved in 275 patients (86% of all study patients) and NEWS at 24 hours in 202 patients (75% of admitted patients). Respiratory rate was the most often missing parameter.

**Table 2:** Clinical characteristics of intermediate–high risk acute pulmonary embolism patients at baseline

	*n* = 99 (%)
Demographics	
Age, median (IQR), y	66 (55–73)
Female; *n* (%)	58 (59%)
Comorbidity	
Malignancy; *n* (%)	31 (31%)
Chronic left ventricular failure; *n* (%)	4 (4%)
PE-related characteristics	
Provoked; *n* (%)	51 (52%)
Admission	
Overall hospital; *n* (%)	98 (99%)
ICU/mcu/ccu; *n* (%)	81 (82%)

Abbreviations: ICU, intensive care unit; IQR, interquartile range; *n* , number of patients; PE, pulmonary embolism; y, years.

### NEWS

Baseline NEWS increased as the ESC risk severity classification increased ( Table 3 ). Median baseline NEWS was 4 (IQR 3–6) in intermediate–high risk patients. In contrast, median baseline NEWS was 1.00 (IQR 0–2) in the low-risk patients, 3 (IQR 2–5) in intermediate–low risk patients, and 10.00 (IQR 4–13) in high-risk patients. NEWS was deemed elevated in 52 (52%, *n* = 99) intermediate–high risk patients, and 20 patients (20%) had a single NEWS parameter >3. In the total group of acute PE patients NEWS was deemed high risk in 128 patients (40%, *n* = 318). Of them 20 patients (16%) were classified as high risk, 49 (38%) as intermediate–low risk, and 7 (6%) as low risk according to the ESC/ERS guideline.

**Table 3:** Baseline and 24 hours NEWS for each ESC classification

	Baseline (median, IQR)	24 hours (median, IQR)
Low risk	1 (0–2) ( *n* = 47)	1 (0–3) ( *n* = 22)
Intermediate–low risk	3 (2–5) ( *n* = 115)	3 (2–5) ( *n* = 90)
Intermediate–high risk	4 (3–6) ( *n* = 90)	3 (2–5) ( *n* = 72)
High risk	10 (4–13) ( *n* = 24)	6 (3–12) ( *n* = 19)
Overall	4 (2–6) ( *n* = 275)	3 (2–5) ( *n* = 202)

Abbreviations: IQR, interquartile range; *n* , number of patients; NEWS, National Early Warning Score.

In 69 intermediate–high risk patients, we were able to also calculate NEWS after 24 hours. In 50 of our 69 patients (62%) NEWS improved (median difference −3; IQR −4 to −2) or was stable after 24 hours. NEWS worsened, with a median difference of 3 (IQR 2–4), in a minority of patients (28%, 19/69) ( Table 4 ).

**Table 4:** NEWS trends over 24 hours for each risk classification

	Stable ( *n* , %)	Improved ( *n* , %)	Worsened ( *n* , %)
Low risk ( *n* = 21)	4 (19.0%)	9 (42.9%)	8 (38.1%)
Intermediate–low risk ( *n* = 86)	14 (20.0%)	41 (61.4%)	31 (36.0%)
Intermediate–high risk ( *n* = 69)	7 (10.0%)	43 (62.3%)	19 (27.5%)
High risk ( *n* = 18)	1 (5.5%)	8 (44.4%)	9 (50.0%)
Overall ( *n* = 193)	26 (13.5%)	100 (51.8%)	67 (34.7%)

Abbreviations: *n* , number of patients; NEWS, National Early Warning Score.

### Adverse Outcome

Overall, PE-related mortality was 3% (26 patients). Another three initially normotensive patients (1%) progressed to hemodynamic instability. Of these 29 patients, 15 patients (52%) had high-risk PE, 8 patients (28%) had intermediate–high risk PE, and 6 (21%) had intermediate–low risk PE. Overall median baseline (minimum) NEWS in the patients who met the primary outcome was 8 (IQR 4–12). In the intermediate–high risk patients who met the primary outcome ( *n* = 8), median baseline (minimum) NEWS was 8 (IQR 5–11) as well. The odds of meeting the composite outcome of PE-related death and/or HD instability increased when baseline NEWS was higher. In the elevated NEWS patients, there is a more pronounced increase in odds of meeting the composite primary outcome ( Table 5 ). Overall, 6 of 190 patients who did not have an elevated NEWS met the composite outcome (3.2%); this was 1 of 47 patients (2.0%) with intermediate–high risk PE.

**Table 5:** Odds on the composite outcome of PE-related death and/or HD instability

Baseline NEWS (continuous variable **)**	
**Overall (** ***n*** ** = 318)**	1.5 (95% CI 1.3–1.7)
**Intermediate–high risk patients (** ***n*** ** = 99)**	1.4 (95% CI 1.1–1.9)
**Elevated NEWS (binary variable)**	
**Overall population (** ***n*** ** = 318)**	6.7 (95% CI 2.65–17.02)
**Intermediate–high risk patients (** ***n*** ** = 99)**	7.2 (95% CI 0.85–61)

Abbreviations: CI, confidence interval; HD, hemodynamic; n, number of patients; NEWS, National Early Warning Score.

Out of 43 intermediate–high risk patients who had an improved NEWS after 24 hours 2 met the primary outcome. Only 1 of 19 patients in whom NEWS worsened after 24 hours and none of 7 patients with a stable NEWS met the primary outcome.

NEWS ≥5 test performance for detection of the primary outcome in the overall population had a sensitivity of 78% (95% CI: 73–83%), specificity of 66% (95% CI: 61–72%), negative predictive value (NPV) of 95% (95% CI: 92–97%), and a positive predictive value (PPV) of 27% (95% CI: 22–32%). Test performance of current ESC risk stratification alone in our study population had a sensitivity of 79% (95% CI: 74–83%), specificity of 64% (95% CI: 59–69%), NPV of 97% (95% CI: 95–98%), and a PPV of 18% (95% CI: 14–23%). For the 99 intermediate–high risk patients, NEWS ≥5 had a sensitivity of 82% (95% CI: 74–90%), specificity of 51% (95% CI: 41–61%), NPV of 96% (95% CI: 90–98%), and a PPV of 17% (95% CI: 11–26%).

## Discussion

In this study of acute PE patients, higher baseline NEWS was associated with a higher odds (OR 1.5) on the primary composite outcome of PE-related death and/or HD deterioration. This was true not only for the complete study population but also for the specific subgroup of patients with intermediate–high risk PE. Therefore, our results suggest that adding NEWS to current risk stratification will improve the predictive accuracy and may help more advanced identification of patients who would benefit from close HD monitoring upon hospital admission, possibly contributing to improvement of appropriate and effective care.

We deemed a NEWS of 5 or above or when a single parameter scores 3 as high risk due to the increased risks of adverse outcomes following recommendations of the National Health Service (NHS). 19 In a subanalysis focused on intermediate–high risk patients only, a high-risk baseline NEWS was indeed associated with a higher risk of developing HD deterioration or death. Our results therefore suggest that it could be considered to abandon expensive close monitoring in intermediate–high risk patients with a low-risk NEWS (baseline <5 and no single parameter >3). If so, in our population, 47 intermediate–high risk patients (47%) would not have been admitted to a high-care department. Of them, only one patient (2%) died suddenly after having a stable low risk NEWS for over 48 hours, pointing to a negative predictive value of 98% for the outcome of PE-related death.

In a recently published analysis from another Dutch cohort of HD stable PE, NEWS, at a threshold of 3, had a 100% sensitivity and 52% specificity for 30-day mortality. 20 We used a higher threshold of 5, which resulted in a lower sensitivity (79%), but a higher specificity (64%). Notably, the study of Bavalia et al focused on determining a safe NEWS threshold to consider home treatment, resulting in higher demands on sensitivity, whereas in our study we aimed to improve identification of patients requiring high-care admission. 20

If we were to assume that high-care admission and close monitoring would prevent further HD decompensation or death, our results indicate that using an elevated NEWS in intermediate–high risk patients would give a number needed to admit to prevent one adverse event (dead or HD decompensation) of 7.4. Based on our results, current ESC/ERS risk stratification alone would lead to 12.4 admissions to prevent one adverse event in the intermediate–high risk group. However, there is no strong evidence for our assumption that close monitoring would prevent death in all circumstances. Using NEWS to guide high-care admissions would thus lead to considerable efficiency improvement of use of scarce resources. Of course, close monitoring, as recommended in the ESC/ERS guideline, does not necessarily mean high-care admission. Frequent evaluation of vital parameters may be sufficient. Also, modern strategies with, for example, in-hospital wearables could be less expensive and may be a good alternative in the near future. 21

We hypothesized that improvement of NEWS over the first 24 hours would be associated with a lower risk of HD deterioration and/or death. Unfortunately, we were not able to retrieve 24-hour NEWS in 125 cases (30 intermediate–high risk, 39% of our study population). In 22 cases (6 intermediate–high risk), the patient was already deceased within 24 hours. The other 104 patients were already discharged or transferred to another hospital. Therefore, we have insufficient data to answer this research question.

## Limitations and Strengths

The main strength of this study is that we combined NEWS with the currently used risk stratification, giving us insight into the value of NEWS in intermediate–high risk PE patients, a heterogenic population for which optimal observation and treatment is not clearly defined at this moment. However, the retrospective design has inherent limitations. The sample size of 99 patients with intermediate–high risk PE at a single center leads to wide confidence intervals, limits generalizability, and lessens certainty about accuracy. We could not retrieve parameters to calculate NEWS in all patients and we had to use minimal NEWS for our analysis, leading to possible underestimating of the predictive value of NEWS. Also, in this retrospective analysis, not all intermediate–high risk patients were admitted for close monitoring. The decision to closely monitor was left to the treating physicians and their argumentations to waive high-care admission could not be retrieved. Therefore, benefit of close monitoring on survival cannot be inferred from this data.

## Conclusion

Adding NEWS to the ESC/ERS risk stratification of acute PE 2 may improve the accuracy of identifying patients with a higher risk of adverse outcomes and may guide the decision to monitor a patient in a high-care department, especially in patients with intermediate–high risk PE.

## Notes

**What is Known About This Topic?**

- Acute PE has an heterogenous clinical outcome.
- ESC guidelines advise risk stratification using RV/LV ratio on CTPA and troponin to determine risk of HD deterioration and/or death. However, this stratification seems not optimal in terms of appropriate and effective care.

**What Does This Paper Add?**

- NEWS is an easy and effective score to evaluate risk of HD deterioration and/or death in acute PE patients.

## Notes

**Corrigendum** : The article has been updated as per Corrigendum published on December 09, 2025 (doi: 10.1055/a-2758-6102).
