# Corrigendum: National Early Warning Score for Predicting Clinical Outcome of Acute Pulmonary Embolism in Intermediate–High Risk Patients

**DOI:** 10.1055/a-2758-6102

**Published:** 2025-12-09

**Authors:** Audrey J. C. Overgaauw, Esther J. Nossent, Lilian J. Meijboom, Erik H. Serne, Yvo M. Smulders, Prabath W. B. Nanayakkara, Harm Jan Bogaard, Pieter Roel Tuinman, Frederikus A. Klok

**Affiliations:** 1 Department of Internal Medicine, Amsterdam UMC, Vrije Universiteit Amsterdam, Amsterdam, The Netherlands; 2 Department of Pulmonary Medicine, Amsterdam UMC, Vrije Universiteit Amsterdam, Amsterdam, The Netherlands; 3 Amsterdam Cardiovascular Sciences Research Institute, Amsterdam UMC, Amsterdam, The Netherlands; 4 Department of Radiology and Nuclear Medicine, Amsterdam UMC, Vrije Universiteit Amsterdam, Amsterdam, The Netherlands; 5 Department of Intensive Care Medicine, Amsterdam UMC, Vrije Universiteit Amsterdam, Amsterdam, The Netherlands; 6 Department of Medicine – Thrombosis and Hemostasis, Leiden UMC, Leiden, The Netherlands

It has been brought to the publisher's attention that the name of the last author Frederikus A. Klok was incorrectly mentioned as Erik A. Klok in the above article published in *TH Open* , Volume 9, a27199061, on November 04, 2025 (doi:  ). The given name has now been corrected in the article as well.

